# Neurovascular decoupling: An early indicator of cognitive decline

**DOI:** 10.1177/13872877261427735

**Published:** 2026-03-13

**Authors:** Suzanne E. van Dijk, Nadieh Drenth, Anne Hafkemeijer, Isa Draaijer, Gerda Labadie, Marie-Noëlle W. Witjes-Ané, Gerard J. Blauw, Serge A. R. B. Rombouts, Jeroen van der Grond, Sanneke van Rooden

**Affiliations:** 1 Department of Radiology, 4501 Leiden University Medical Center, Leiden, The Netherlands; 2 Institute of Psychology, Leiden University, Leiden, The Netherlands; 3 Leiden Institute for Brain and Cognition, Leiden, The Netherlands; 4 Department of Geriatrics and department of Psychiatrics, 4501 Leiden University Medical Center, Leiden, the Netherlands; 5 Department of Internal Medicine, Section of Gerontology and Geriatrics, 4501 Leiden University Medical Center, Leiden, the Netherlands; 6 Department of Geriatrics, Haaglanden Medical Center, The Hague, the Netherlands

**Keywords:** Alzheimer's disease, BOLD fMRI, cerebrovascular dysfunction, cognitive decline, mild cognitive impairment, MRI markers, neurovascular coupling, small vessel disease, subjective cognitive impairment, visual stimulation paradigm

## Abstract

**Background:**

Cognitive decline in Alzheimer's disease (AD) is increasingly linked to cerebrovascular dysfunction, including small vessel disease (SVD). Conventional SVD MRI markers primarily detect late-stage structural damage. Neurovascular coupling (NVC) has emerged as an early indicator of cerebrovascular function and is cross-sectionally associated with cognition, but its association with longitudinal decline remains unclear.

**Objective:**

To investigate whether baseline NVC is associated with cognitive decline over 3.5 years in individuals ranging from no cognitive complaints to memory clinic patients with subjective cognitive impairment (SCI) or mild cognitive impairment (MCI).

**Methods:**

Seventy-five participants underwent cognitive assessment and MRI, including BOLD fMRI during visual stimulation to assess NVC. Cognitive decline was analyzed as dichotomous outcome (stable versus decline; n = 75) using logistic regression and as continuous outcome for domain-specific change (z-score change; n = 69) using linear regression.

**Results:**

Lower baseline NVC was associated with global cognitive decline, both dichotomously (OR = 42.29, p = 0.017) and continuously (B = 0.45, p = 0.012). Baseline NVC was also associated with a decline in executive function (B = 0.47, p = 0.043), but not with other domain-specific decline. Among conventional SVD MRI markers, only lacunar infarcts were associated with decline (OR = 4.12, p = 0.037).

**Conclusions:**

Baseline NVC appears to be a predictor of global cognitive decline, outperforming most conventional SVD MRI markers. These findings support the potential utility of NVC as a prognostic marker of cognitive decline in early-stage AD, highlighting its promise for early intervention strategies targeting cerebrovascular dysfunction.

## Introduction

Cognitive decline is a hallmark of neurodegenerative diseases, prominently featured in Alzheimer's disease (AD). Traditionally, cognitive decline in AD has been attributed to pathological processes leading to neurodegeneration, including the accumulation of amyloid-β (Aβ) plaques and tau neurofibrillary tangles.^1^ However, emerging evidence highlights a critical role for cerebrovascular dysfunction in the onset and progression of AD.^2,3^

Cerebral small vessel disease (SVD), a pathological process affecting the small arteries, arterioles, capillaries, and venules, is highly prevalent in older adults and frequently co-occurs with AD.^4^ SVD is associated with worse cognitive performance in patients with AD,^5,6^ in memory clinic populations,^7–9^ and even in cognitively healthy older adults, where it predicts both future cognitive decline and risk of dementia.^5,7–10^ SVD is typically assessed with structural magnetic resonance imaging (MRI) using markers such as white matter hyperintensities (WMH), cerebral microbleeds, lacunar infarcts, and dilated perivascular spaces (DPVS). These markers provide valuable insight into cerebrovascular damage. However, they largely reflect late-stage, irreversible pathology and may be insensitive to earlier, potentially modifiable vascular changes.

In this respect, neurovascular coupling (NVC), the process by which neural activity elicits localized increases in cerebral blood flow to meet metabolic demands, has emerged as a promising early indicator of microvascular health.^11,12^ Importantly, changes in NVC can occur before structural brain damage becomes detectable on conventional MRI.^13^ Impaired NVC has been observed in SVD,^14^ cerebral amyloid angiopathy (CAA),^15–17^ and in AD and its prodromal stages.^18,19^ These alterations appear clinically relevant, as NVC correlates with cognitive performance, both in healthy older adults^20^ and in AD and its early stages.^18^ Preclinical studies further support a causal role as pharmacological disruption of NVC impairs memory,^21^ while interventions restoring NVC improve cognition.^22^

Blood-oxygenation-level-dependent (BOLD) functional MRI (fMRI) provides a non-invasive approach to assess NVC by detecting the hemodynamic response to neuronal activity. Visual stimulation paradigms are particularly robust for this purpose and have demonstrated altered NVC responses, even in the presymptomatic phase of Dutch-type hereditary CAA (D-CAA).^17^ Such alterations appear before structural abnormalities are detectable with conventional MRI, underscoring the potential of BOLD fMRI in response to visual stimulation to capture early vascular alterations.

Despite increasing evidence for vascular contributions to cognitive decline in the disease process of AD, important knowledge gaps remain. It is unknown whether impaired NVC is associated with future cognitive decline and how this compares to conventional SVD MRI markers. To address this, we conducted a 3.5-year longitudinal study including participants without cognitive complaints and memory clinic patients with subjective cognitive impairment (SCI) and mild cognitive impairment (MCI).We investigated whether baseline NVC is associated with cognitive decline both (1) globally and (2) across specific cognitive domains, and we compared these associations with those of established SVD MRI markers. We hypothesized that lower baseline NVC would be associated with greater cognitive decline.

## Methods

### Study design and participants

This longitudinal study was conducted at the Department of Radiology, Leiden University Medical Center (Leiden, Netherlands) from September 2023 to June 2024, with the baseline phase between September 2019 and December 2021.^18^ At baseline, the cohort comprised memory clinic patients diagnosed with SCI or MCI, as well as cognitively healthy individuals (i.e., no cognitive complaints and no history of memory clinic visits). Memory clinic patients were recruited from the memory clinics of the Leiden University Medical Center (Leiden, Netherlands) and the Haaglanden Medical Center (The Hague, Netherlands). Patients were referred by their general practitioner or a medical specialist due to cognitive complaints and underwent a standardized diagnostic protocol at the memory clinic, including medical and neurological examination, neuropsychological assessment, and brain imaging (CT or MRI). Fluid biomarkers and advanced imaging techniques such as amyloid PET were not part of the diagnostic work-up. Diagnoses were made in multidisciplinary consensus meetings using National Institute of Neurological and Communicative Disorders and Stroke and the Alzheimer's Disease and Related Disorders Association (NINCDS-ADRDA) criteria.^23^ MCI was defined as objective cognitive deficits with preserved daily functioning, whereas SCI was defined as the presence of cognitive complaints in the absence of objective cognitive deficits.

Cognitively healthy participants without cognitive complaints were recruited separately through various advertisements. These individuals were excluded if neuropsychological assessment revealed cognitive deficits. Additional exclusion criteria for all participants included Mini-Mental State Examination (MMSE) < 19, inability to provide informed consent, age > 90 years, contraindications to MRI or performing a visual task during MRI, seizure withing the prior year, non-correctable visual impairment, and presence of any other primary neurological or neurodegenerative disorder (e.g., Parkinson's disease)).

At baseline, participants underwent an MRI scan of the brain on the same 3-Tesla scanner, medical and neurological screening, and a neuropsychological assessment. For the follow-up study, participants with baseline MRI data were re-invited for follow-up approximately 3.5 years later. Follow-up procedures replicated those performed at baseline; however, only neuropsychological data were included in the present analyses, and follow-up MRI data were not utilized. Attrition and reasons for loss to follow-up are detailed in Supplemental Figure I. The study protocol was approved by the Medical Ethics Committee of Leiden Den Haag Delft (Ethics Code: NL83653.058.23). All participants provided written informed consent, and the study adhered to institutional guidelines and the principles of the Declaration of Helsinki.

### Data collection

*Image acquisition.* All imaging was performed on the same 3-Tesla scanner (Achieva, Philips Medical Systems, Eindhoven, Netherlands) using a 32-channel head coil. Acquired sequences included three-dimensional (3D) T1-weighted, T2-weighted, T2*-weighted, 3D fluid-attenuated inversion recovery (FLAIR), and visually stimulated BOLD fMRI scans. Full acquisition parameters have been described previously.^18^

The BOLD fMRI scans were collected during a block-design visual stimulation task, using alternating sequence of an 8 Hz flashing black-and-white radial checkerboard and a uniform gray screen, as detailed in a prior study.^16^ Each condition lasted 20 and 28 s, respectively, with four stimulus-rest cycles per scan. A red fixation dot was displayed throughout the task and intermittently changed color (light red to dark red). Participants were instructed to respond via button press whenever they detected the color change, ensuring engagement and visual attention.

*Image analysis and processing.* All MRI scans were visually inspected for quality assurance to confirm the absence of significant artifacts. Cerebral microbleeds were identified on T2*-weighted images and classified as lobar or deep, following established diagnostic criteria and anatomical definitions based on the Boston criteria.^24,25^ Lacunar infarcts were defined as small, round, or ovoid lesions (3–15 mm in diameter), appearing hyperintense on T2-weighted images and showing a central hypointensity with a hyperintense rim on FLAIR.^26^ Dilated perivascular spaces (DPVS) were rated separately in the basal ganglia (DPVS-BG) and centrum semiovale (DPVS-CSO) on axial T2-weighted images in accordance with STRIVE criteria.^27^ DPVS-BG were graded using a 4-point scale,^28^ and DPVS-CSO were scored using a 5-point scale.^29^ WMHs were quantified semi-automatically on FLAIR images using a previously validated segmentation method.^30^ To assess NVC, BOLD fMRI data acquired during visual stimulation were analyzed to extract the BOLD signal amplitude from the occipital lobe in response to the checkerboard paradigm, as previously described.^16^ BOLD amplitude was used as the primary NVC outcome and was preferred over other temporal metrics such as time to peak or time to baseline.^31^ Higher BOLD amplitude values reflect better NVC.

*Neuropsychological assessment.* All participants performed standardized neuropsychological testing. The MMSE^32^ was used as a measure of global cognitive functioning. Memory was evaluated using the Visual Association Test (VAT)^33^ and the 8-words test.^34^ Psychomotor speed was assessed using Trail Making Test (TMT) Part A^35^ and the D-KEFS Color Word Test Part 1 and 2.^36^ For testing of executive function, TMT Part B and D-KEFS Color Word Test Part 3 and 4^36^ were used. Language was evaluated using letter and category fluency^37,38^ and the Boston Naming Test (BNT).^39^ Raw scores from baseline and follow-up assessments were converted to z-scores using the baseline mean and standard deviation. A global cognition composite score was calculated by averaging z-scores across all tests, and domain-specific scores for memory, executive functioning, psychomotor speed, and language were derived by averaging z-scores within each domain. Scores on the TMT and D-KEFS Color Word Test were inverted so that higher scores consistently indicated better performance.

*Cognitive decline.* For our primary objective, global cognitive decline was defined as a dichotomous outcome (cognitive decline vs. cognitively stable). Cognitive decline was determined using one of three approaches, depending on participant's ability to participate in the follow-up study:

Participants with follow-up assessment:

1. **Diagnostic conversion:** Participants were classified as cognitive decliners if they progressed to a more severe cognitive stage, i.e., SCI to MCI or MCI to AD-related dementia, based on memory clinic assessment during the follow-up period.
2. **Neuropsychological assessment (NPA) (distribution-based threshold)**: Decline was also defined as a reduction of at least 0.5 standard deviations (−0.5 SD) from baseline global cognition composite score. This cut-off was selected because change of 0.4–0.5 SD is widely considered clinically meaningful in cognitive aging and dementia research.^40^

Participants without follow-up assessment:

3.  **Family-reported severe decline or institutionalization:** Cognitive decline for these participants was determined based on family reports of significant functional impairment due to cognitive deterioration or admission to a psychogeriatric ward.

For the secondary objective, analyses were restricted to participants with follow-up assessment. Cognitive decline was treated as a continuous outcome, defined as the change in z-scores from baseline to follow-up for each cognitive domain (memory, executive function, psychomotor speed, and language) as well as for a global cognition composite score derived from all cognitive tests (Δ = follow-up – baseline).

### Covariates

Covariates included age, sex, cardiovascular risk factors, and history of stroke. Cardiovascular risk factors comprised hypertension, hyperlipidemia, and diabetes defined as self-reported or medication use. Smoking status was classified as current smoker. History of stroke included both ischemic and hemorrhagic events.

### Statistical analysis

Baseline demographic and clinical characteristic were compared between participants with and without cognitive decline using independent t-tests for normally distributed continuous variables, Mann-Whitney U tests for skewed variables, and χ^2^ tests for categorical variables; Fisher's exact test was used when expected cell counts were < 5. Continuous variables are presented as mean ± SD (range), and categorical variables as counts and percentages.

MRI markers included in the analyses were BOLD amplitude, lobar and deep microbleeds, lacunar infarcts, DPVS in the CSO and BG, and WMHs. Microbleeds and lacunar infarcts were dichotomized as present versus absent, whereas DPVS were categorized as high (scores 3–4) versus low (scores 0–2). WMH volumes were log-transformed to reduce skewness.

For the primary objective, logistic regression was used to examine whether baseline BOLD amplitude was associated with cognitive decline (dichotomous), with age, sex, hypertension, hyperlipidemia, diabetes mellitus, current smoking, and history of ischemic and hemorrhagic stroke included as covariates. In contrast to the linear models, time interval was not included as a covariate in the dichotomous cognitive decline models because the decline status for part of the decline group was determined through family report, institutionalization, or new clinical diagnosis during the follow-up period and was therefore not tied to the exact length of follow-up. To compare BOLD amplitude with conventional SVD MRI markers, logistic regression models were run for each marker separately. Associations were summarized as odds ratios (OR) with 95% confidence intervals (CIs) and visualized in a forest plot. For significant predictors, discriminative ability was evaluated using the area under the receiver operating characteristic curve (AUC).

For the secondary objective, multivariable linear regression models were used to examine the association between baseline BOLD amplitude and change in domain-specific and global cognitive performance (continuous), with age, sex, hypertension, hyperlipidemia, diabetes mellitus, current smoking, history of ischemic and hemorrhagic stroke, and time interval between assessments included as covariates. Separate models were run for each conventional SVD MRI marker. Moreover, to provide additional insight, cross-sectional analyses at baseline were conducted using the same regression framework, with baseline cognitive scores as dependent variables and MRI markers as independent, with age and sex, hypertension, hyperlipidemia, diabetes mellitus, current smoking, history of ischemic and hemorrhagic stroke included as covariates. Associations were reported as unstandardized regression coefficients (B) with 95% CIs, standardized regression coefficients (β) and p-values. Additionally, Pearson's correlation were calculated between baseline BOLD amplitude and both baseline cognitive scores and cognitive change scores. Corresponding scatterplots with R-values are provided.

All statistical tests were two-tailed significance set at α = 0.05. Given the exploratory nature and interrelatedness of outcomes, no correction for multiple comparison was applied, and findings are interpreted accordingly. Analyses were performed using Statistical Package of Social Sciences (SPSS) version 29.0 (IBM Corp., Armonk NY).

## Results

### Participant inclusion

The participant inclusion process is illustrated in Supplemental Figure I. At baseline, 108 individuals were enrolled. During follow-up, three participants died, six were unable to participate due to severe cognitive decline or nursing home admission, seven had severe somatic conditions, nine declined further participation, six were lost to follow-up for other or unknown reasons, and five did not respond. Additionally, two participants without baseline MRI data were not invited to follow-up. This resulted in 70 participants taking part in the follow-up study. One of these participants did not complete follow-up neuropsychological testing and could not be classified as cognitively stable or as having cognitive decline; this individual was excluded from analyses.

For the primary analysis, the sample included 69 participants with complete baseline and follow-up data, plus 6 additional participants (two SCI and four MCI patients) identified as having family-reported cognitive decline; these six participants did not undergo the follow-up assessment, resulting in a total of 75 participants. For the secondary analyses, only the 69 participants with complete baseline and follow-up data were included.

### Demographic and clinical characteristics

Baseline characteristics of the total sample and participants stratified by cognitive outcome are presented in Table 1. Fifty-one participants remained cognitively stable and 24 were classified as cognitive decliners over the 3.5-year follow-up period (*M* = 3 years 4 months, *SD* ± 6 months), follow-up duration did not differ significantly between the cognitive stable and cognitive decline group (3 years 5 months ± 6 months versus 3 years 3 months ± 6 months, p = 0.214). Participants in the cognitive decline group were significantly older than those in the cognitively stable group (74.9 ± 8.0 versus 67.0 ± 7.9 years, p < 0.001) and had a lower baseline MMSE score (27.5 ± 1.9 versus 28.7 ± 1.4, p = 0.010). Sex distribution did not differ significantly between groups. Diagnosis at baseline differed between the cognitive stable and cognitive decline group (p < 0.001). The cognitively stable group predominantly consisted of individuals without cognitive complaints at baseline (62.7%), whereas the cognitive decline group had a higher proportion of participants with MCI at baseline (58.3%). Cardiovascular risk factors, including hypertension, hyperlipidemia, diabetes mellitus, and current smoking, were similar between groups, with no statistically significant differences observed (p > 0.05 for all). Medication use (antihypertensive or cholesterol-lowering) also did not differ. History of ischemic or hemorrhagic stroke did not differ between the cognitive stable and cognitive decline group.

**Table 1.** Demographic and clinical characteristics at baseline of the total sample and cognitively stable and cognitive decline subgroups.

	Total sample	Cognitively stable	Cognitive decline	*p*
N	75	51	24	
Cognitive decline method				
Converter	n/a	n/a	4	-
NPA	n/a	n/a	14	-
Family reported	n/a	n/a	6	-
Follow-up duration	3y4 m ± 6 m (2y1 m – 4y4 m)	3y5 m ± 6 m (2y3 m – 4y4 m)	3y3 m ± 6 m (2y1 m – 4y1 m)	0.214
Demographics				
Age	69.5 ± 8.7 (52–86)	67.0 ± 7.9 (52–86)	74.9 ± 8.0	<0.001
Sex female	30 (40.0%)	24 (47.1%)	6 (25.0%)	0.069
MMSE score	28.3 ± 1.6 (24–30)	28.7 ± 1.4 (24–30)	27.5 ± 1.9 (24–30)	0.010
Diagnosis at baseline				<0.001
No cognitive complaints	36 (48.0%)	32 (62.7%)	4 (16.7%)	
SCI	19 (25.3%)	13 (25.5%)	6 (25.0%)	
MCI	20 (26.7%)	6 (11.8%)	14 (58.3%)	
Cardiovascular risk factors				
Hypertension	29 (38.7%)	18 (35.3%)	11 (45.8%)	0.307
Hyperlipidemia	21 (28.0%)	12 (23.5%)	9 (37.5%)	0.203
Diabetes Mellitus	8 (10.7%)	5 (9.8%)	3 (12.5%)	0.705
Smoking (current)	7 (9.3%)	7 (13.7%)	0 (0.0%)	0.144
Medication				
Antihypertensive	24 (32.0%)	15 (29.4%)	9 (37.5%)	0.484
Cholesterol lowering	17 (22.7%)	10 (19.6%)	7 (29.2%)	0.356
History of vascular disease				
Ischemic stroke	3 (4.0%)	1 (2.0%)	2 (8.3%)	0.238
Hemorrhagic stroke	2 (2.7%)	1 (2.0%)	1 (4.2%)	0.546
Neurovascular coupling				
BOLD amplitude (%)	1.06 ± 0.27 (0.31–1.80)	1.14 ± 0.25 (0.69–1.80)	0.89 ± 0.25 (0.31–1.39)	<0.001
SVD MRI markers				
≥ 1 Lobar microbleeds	33 (44.0%)	22 (43.1%)	11 (45.8%)	0.826
≥ 1 Deep microbleeds	8 (10.7%)	6 (11.8%)	2 (8.3%)	0.653
≥ 1 Lacunar infarcts	22 (29.3%)	10 (19.6%)	12 (50.0%)	0.005
DPVS-CSO – high count	43 (57.3%)	31 (60.8%)	12 (50.0%)	0.951
DPVS-BG – high count	30 (40.0%)	18 (35.3%)	12 (50.0%)	0.225
WMHs cm3	5.67 ± 5.65 (0.53–23.68)	4.72 ± 4.58 (0.65–17.96)	7.68 ± 7.14 (0.53–23.68)	0.104

BOLD: blood-oxygen-level-dependent; DPVS-BG: dilated perivascular spaces in the basal ganglia; DPVS-CSO: dilated perivascular spaces in the centrum semiovale;; MCI: mild cognitive impairment; MMSE: Mini-Mental State Examination; NPA: neuropsychological assessment; MRI: magnetic resonance imaging; SCI: subjective cognitive impairment; SVD: small vessel disease; WMHs: white matter hyperintensities. Values are presented as mean ± SD (range) for continuous variables, or number (percentage) for categorical variables. To compare groups (cognitively stable versus cognitive decline) independent t-tests were used for normally distributed continuous variables, Mann-Whitney U tests for non-normally distributed continuous variables, and chi-square (χ^2^) tests for categorical variables. When the expected cell counts were small (i.e., <5 in >20% of cells), Fisher's Exact Test was used instead of χ^2^. p-values are reported. **Missing data.** Follow-up duration (n = 6), Hypertension (n = 1), hyperlipidemia (n = 3), history of hemorrhagic stroke (n = 1), lacunar infarcts (n = 1), and DPVS-CSO (n = 4).

Baseline BOLD amplitude was significantly lower in the cognitive decline group (0.89 ± 0.25 versus 1.14 ± 0.25, p < 0.001). The prevalence of baseline lacunar infarct was significantly higher in the cognitive decline group (50.0% versus 19.6%, p = 0.005). Other baseline SVD MRI markers did not differ between the groups.

### Cognition

Table 2 summarizes baseline and follow-up cognitive performance (z-scores) as well as the cognitive change scores for the total sample as well as the diagnostic subgroups. Diagnostic subgroups are presented for descriptive purposes only; all regression analyses were conducted on the total sample. As expected, baseline cognitive performance was highest in the no cognitive complaints group and lowest in the MCI group. A similar pattern was observed for cognitive change scores, with the smallest decline in the no cognitive complaints group and the largest in the MCI group.

**Table 2.** Cognitive performance (z-scores) at baseline and follow-up, and change over time, for the total sample and diagnostic subgroups.

	Total sample	No cognitive complaints	SCI	MCI
N baseline	75	36	19	20
N follow-up	69	36	17	16
Global cognition				
Baseline	0.00 ± 0.68 (−1.89–1.18)	0.39 ± 0.28 (−0.20–0.89)	0.14 ± 0.60 (−0.89–1.18)	−0.85 ± 0.50 (−1.89 – −0.22)
Follow-up	−0.24 ± 0.87 (−2.74–1.05)	0.20 ± 0.36 (−0.71–0.91)	−0.09 ± 0.79 (−1.97–1.05)	−1.38 ± 0.76 (−2.74 – −0.39)
Change (Δ)	−0.32 ± 0.37 (1.60–0.39)	−0.20 ± 0.24 (−0.69–0.37)	−0.31 ± 0.37 (−1.11–0.15)	−0.61 ± 0.47 (−1.60–0.39)
Memory				
Baseline	0.00 ± 0.80 (−2.72–0.90)	0.42 ± 0.39 (−0.66–0.90)	0.21 ± 0.49 (−0.71–0.90)	−0.97 ± 0.79 (−2.72–0.15)
Follow-up	−0.40 ± 1.13 (−3.82–0.90)	0.17 ± 0.47 (−1.04–0.90)	−0.21 ± 0.93 (−2.69–0.75)	−1.89 ± 1.08 (−3.82 – −0.13)
Change (Δ)	−0.46 ± 0.62 (−2.66–0.87)	−0.25 ± 0.46 (−1.79–0.87)	−0.47 ± 0.69 (−2.20–0.36)	−0.93 ± 0.62 (−2.66–0.09)
Executive function				
Baseline	0.00 ± 0.86 (−3.12–1.08)	0.45 ± 0.36 (−0.43–1.08)	0.04 ± 0.67 (−1.57–0.91)	−0.81 ± 1.06 (−3.12–0.65)
Follow-up	−0.07 ± 0.97 (−3.26–1.03)	0.31 ± 0.47 (−0.68–1.03)	−0.05 ± 0.95 (−2.74–1.00)	−0.93 ± 1.30 (−3.26–0.67)
Change (Δ)	−0.18 ± 0.48 (−1.84–0.71)	−0.14 ± 0.31 (−1.06–0.43)	−0.18 ± 0.41 (−1.16–0.63)	−0.29 ± 0.79 (−1.84–0.71)
Psychomotor speed				
Baseline	0.01 ± 0.81 (−2.40–1.32)	0.38 ± 0.48 (−0.81–1.11)	0.22 ± 0.81 (1.63–1.32)	−0.84 ± 0.67 (−2.40–0.25)
Follow-up	−0.19 ± 1.11 (−4.84–1.24)	0.23 ± 0.63 (−1.11–1.23)	0.02 ± 0.86 (−1.75–1.24)	−1.38 ± 1.39 (−4.84–0.35)
Change (Δ)	−0.27 ± 0.62 (−3.53–1.02)	−0.15 ± 0.46 (−1.53–0.69)	−0.25 ± 0.31 (−0.73–0.34)	−0.57 ± 1.02 (−3.53–1.02)
Language				
Baseline	0.00 ± 0.76 (−2.21–1.60)	0.37 ± 0.44 (−0.38–1.44)	0.08 ± 0.75 (−1.10–1.60)	−0.75 ± 0.69 (−2.21–0.28)
Follow-up	−0.20 ± 0.81 (−2.05–1.63)	0.09 ± 0.51 (−0.79–1.55)	−0.05 ± 0.96 (−1.59–1.63)	−1.02 ± 0.68 (−2.05–0.12)
Change (Δ)	−0.29 ± 0.40 (−1.04–0.96)	−0.29 ± 0.40 (−1.04–0.75)	−0.21 ± 0.45 (−0.99–0.96)	−0.38 ± 0.35 (−0.76–0.38)

MCI: mild cognitive impairment; SCI: subjective cognitive impairment. This table is for descriptive purposes; all linear regression analyses for objective 2 were performed on the total sample. Values are presented as mean ± SD (range). Z-scores were standardized using the baseline mean and SD of the total sample. Change scores reflect follow-up minus baseline z-scores, with higher values indicating better performance. **Missing data.** No missing data.

### Primary objective: global cognitive decline (dichotomous)

**Figure 1.**
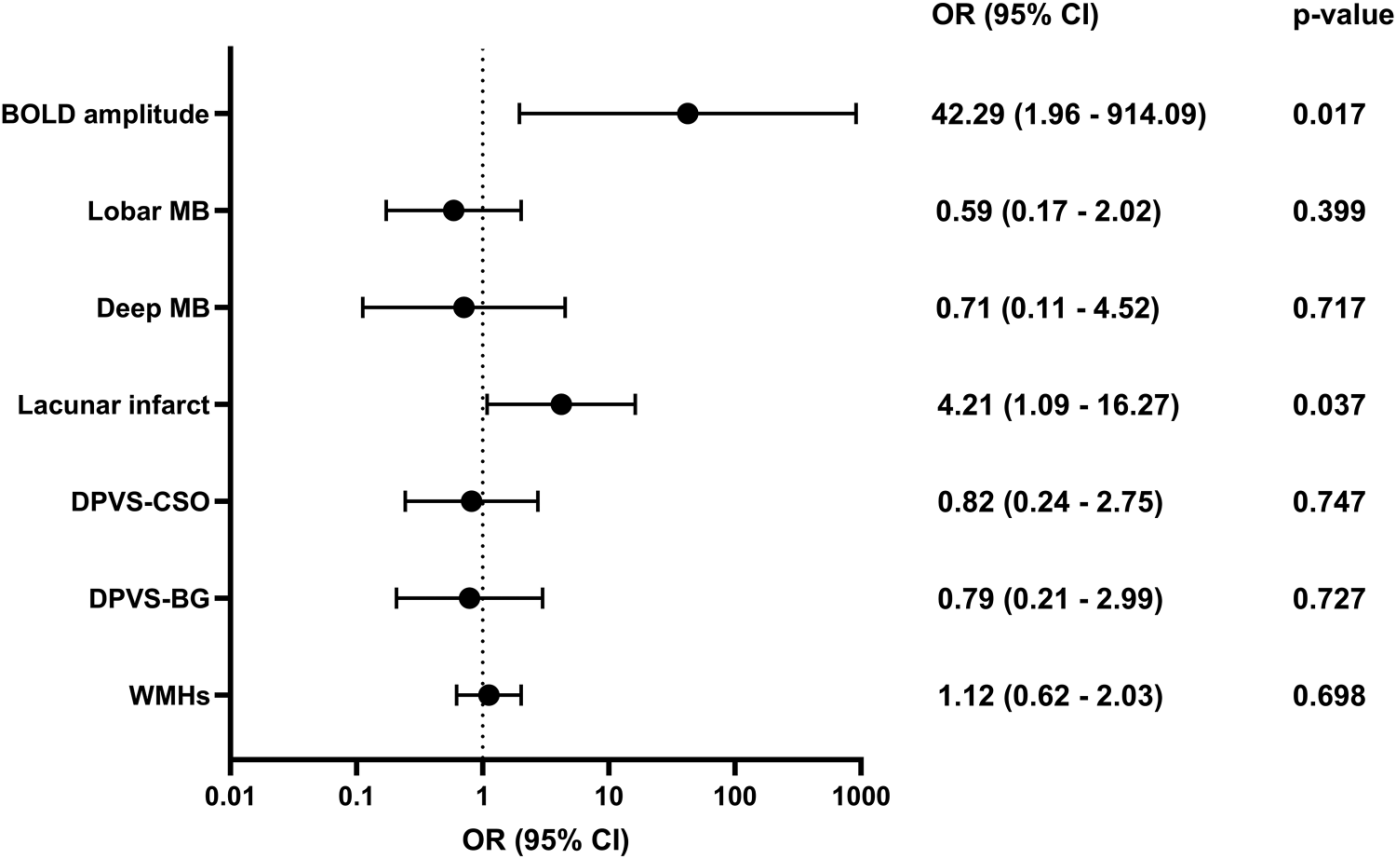
Forest plot showing associations between baseline BOLD amplitude and conventional SVD MRI markers with cognitive decline over 3.5 year. Odd ratios (OR) and 95% confidence intervals (CIs) are presented on a log10. Each row represents an individual predictor, with odds ratios indicated by dots and CIs shown by horizontal lines, with p-values derived from logistic regression analyses. To ensure consistent interpretation, BOLD amplitude scores were reversed (multiplied by −1) so that higher values of both BOLD amplitude and SVD MRI markers indicate worse outcomes, aligning the directionality of all predictors with greater risk of cognitive decline.

**Figure 2.**
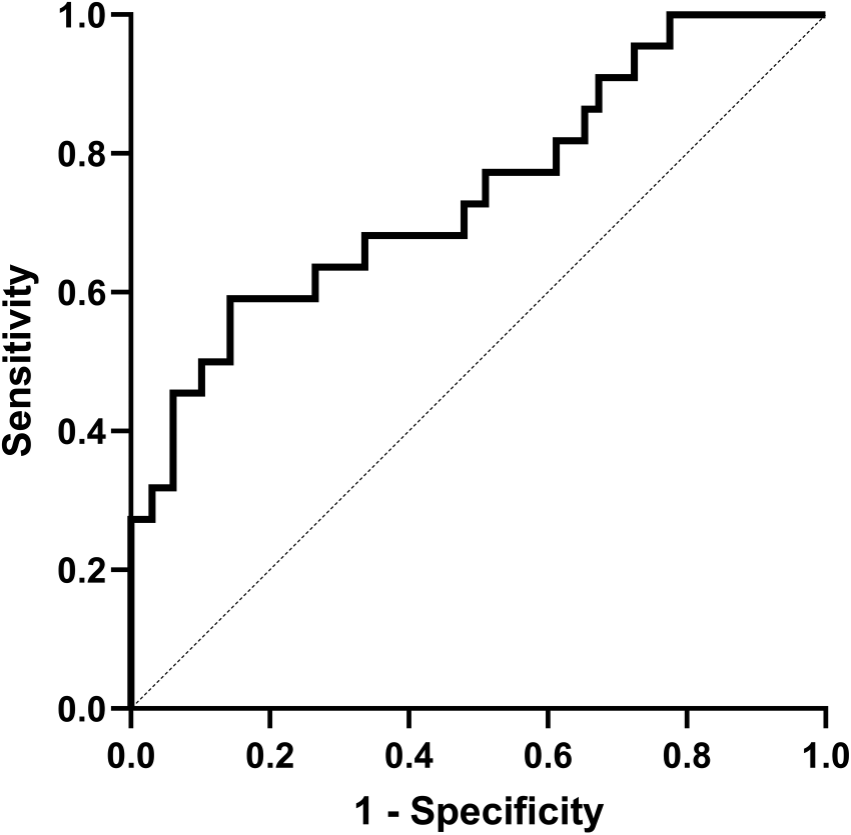
Receiver Operating Characteristic (ROC) Curve for BOLD amplitude in predicting cognitive decline over 3.5 years. The area under the curve (AUC) was 0.75, indicating moderate discrimination. The ROC illustrates the trade-off between sensitivity (true positive rate) and specificity (1-false positive rate) across different thresholds, suggesting that BOLD amplitude has a fair ability to differentiate between individuals who show cognitive decline and those who will remain cognitively stable.

Multiple logistic regression analyses examined whether baseline BOLD amplitude and conventional SVD MRI markers were associated with global cognitive decline over 3.5 years (Figure 1), adjusted for age, sex, cardiovascular risk factors (hypertension, hyperlipidemia, diabetes, and smoking) and history of stroke. Lower BOLD amplitude at baseline was strongly associated with increased odds of cognitive decline (OR = 42.29, p = 0.017), with moderate discriminative performance (AUC = 0.75; Figure 2). Among conventional SVD markers, only lacunar infarcts were significantly associated with cognitive decline (OR = 4.21, p = 0.037, AUC = 0.66). Other conventional SVD MRI markers, i.e., lobar and deep microbleeds, DPVS, and WMHs, showed no significant association (p > 0.05 for all).

### Cross-sectional associations with domain-specific cognitive performance

**Figure 3.**
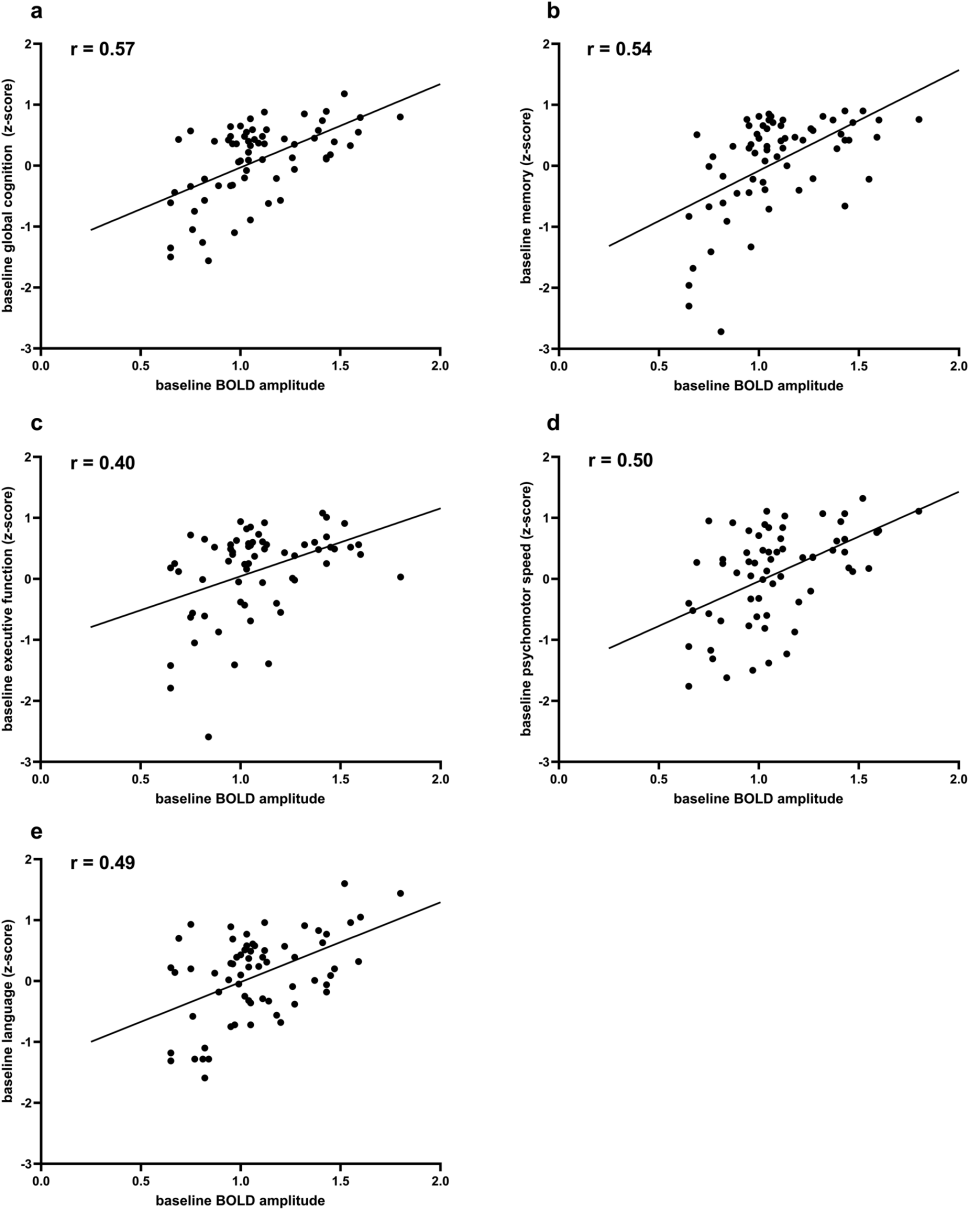
Scatterplots showing the associations between baseline BOLD amplitude and baseline cognitive performance. Pearson's correlation coefficients (r) are displayed in each panel. Lower BOLD amplitude is associated with lower a) global cognition and lower scores across all cognitive domains, i.e., b) memory, c) executive function, d) psychomotor speed, and e) language).

Cross-sectional analyses provided informative associations between baseline MRI markers and baseline cognitive performance (Table 3), adjusted for age, sex, cardiovascular risk factors (hypertension, hyperlipidemia, diabetes, and smoking) and history of stroke. Lower BOLD amplitude was significantly associated with poorer global cognition (p < 0.001) and across all domains, i.e., memory (p < 0.001), executive function (p = 0.042), psychomotor speed (p = 0.003), and language (p < 0.001) (Figure 3). A significant association was found for lobar MB and memory (p = 0.027), but not with other cognitive domains. No significant cross-sectional associations were observed for other conventional SVD MRI markers, including deep microbleeds, lacunar infarcts, DPVS, and WMHs (p > 0.05 for all).

**Table 3.** Cross-sectional associations between baseline MRI markers and baseline cognition z-scores.

	Global cognition (composite score)			Memory			Executive function			Psychomotor speed			Language		
	B (95% CI)	β	p	B (95% CI)	Β	p	B (95% CI)	β	p	B (95% CI)	β	p	B (95% CI)	β	p
BOLD amplitude	0.98 (0.50–1.46)	0.41	**<0** **.** **001**	1.27 (0.57–1.96)	0.42	**<0**.**001**	0.66 (0.02–1.29)	0.24	**0**.**042**	0.95 (0.34–1.56)	0.32	**0**.**003**	1.08 (0.49–1.68)	0.41	**<0**.**001**
Lobar microbleed	−0.11 (−0.39–0.16)	−0.09	0.419	−0.43 (−0.81–−0.05))	−0.27	**0**.**027**	0.08 (−0.27–0.43)	0.05	0.658	0.08 (−0.27–0.43)	0.05	0.652	−0.16 (−0.49–0.18)	−0.11	0.364
Deep microbleed	0.06 (−0.35–0.47)	0.03	0.768	−0.07 (−0.66–0.52)	−0.03	0.807	0.11 (−0.41–0.63)	0.05	0.680	0.11 (−0.41–0.63)	0.05	0.667	0.15 (−0.35–0.66)	0.07	0.547
Lacunar infarct	0.08 (−0.26–0.42)	0.05	0.642	−0.17 (−0.65–0.31)	−0.10	0.478	0.21 (−0.21–0.64)	0.12	0.316	0.19 (−0.23–0.62)	0.11	0.370	0.16 (−0.25–0.57)	0.10	0.444
DPVS-CSO	0.13 (−0.13–0.39)	0.11	0.312	0.08 (−0.30–0.45)	0.05	0.686	0.13 (−0.16–0.43)	0.10	0.360	0.13 (−0.20–0.45)	0.08	0.433	0.15 (−0.18–0.48)	0.11	0.364
DPVS-BG	0.12 (−0.17–0.42)	0.09	0.415	0.16 (−0.26–0.58)	0.10	0.460	0.21 (−0.16–0.58)	0.14	0.259	0.09 (−0.29–0.46)	0.05	0.640	−0.00 (−0.37–0.36)	−0.00	0.983
WMHs	−0.08 (−0.23–0.08)	−0.11	0.324	−0.18 (−0.40–0.03)	−0.21	0.097	−0.06 (−0.25–0.14)	−0.07	0.558	−0.09 (−0.28–0.11)	−0.10	0.372	−0.01 (−0.21–0.18)	−0.02	0.886

B: unstandardized regression coefficient; β: standardized regression coefficient; CI: confidence interval; BOLD: blood-oxygen-level-dependent; DPVS-BG: dilated perivascular spaces in the basal ganglia; DPVS-CSO: dilated perivascular spaces in the centrum semiovale; WMHs: white matter hyperintensities. Cross-sectional associations between baseline MRI markers and baseline cognitive domain z-scores were assessed using linear regression analyses. Significant associations are highlighted in bold (p < 0.05).

### Secondary objective: domain-specific cognitive decline (continuous)

**Figure 4.**
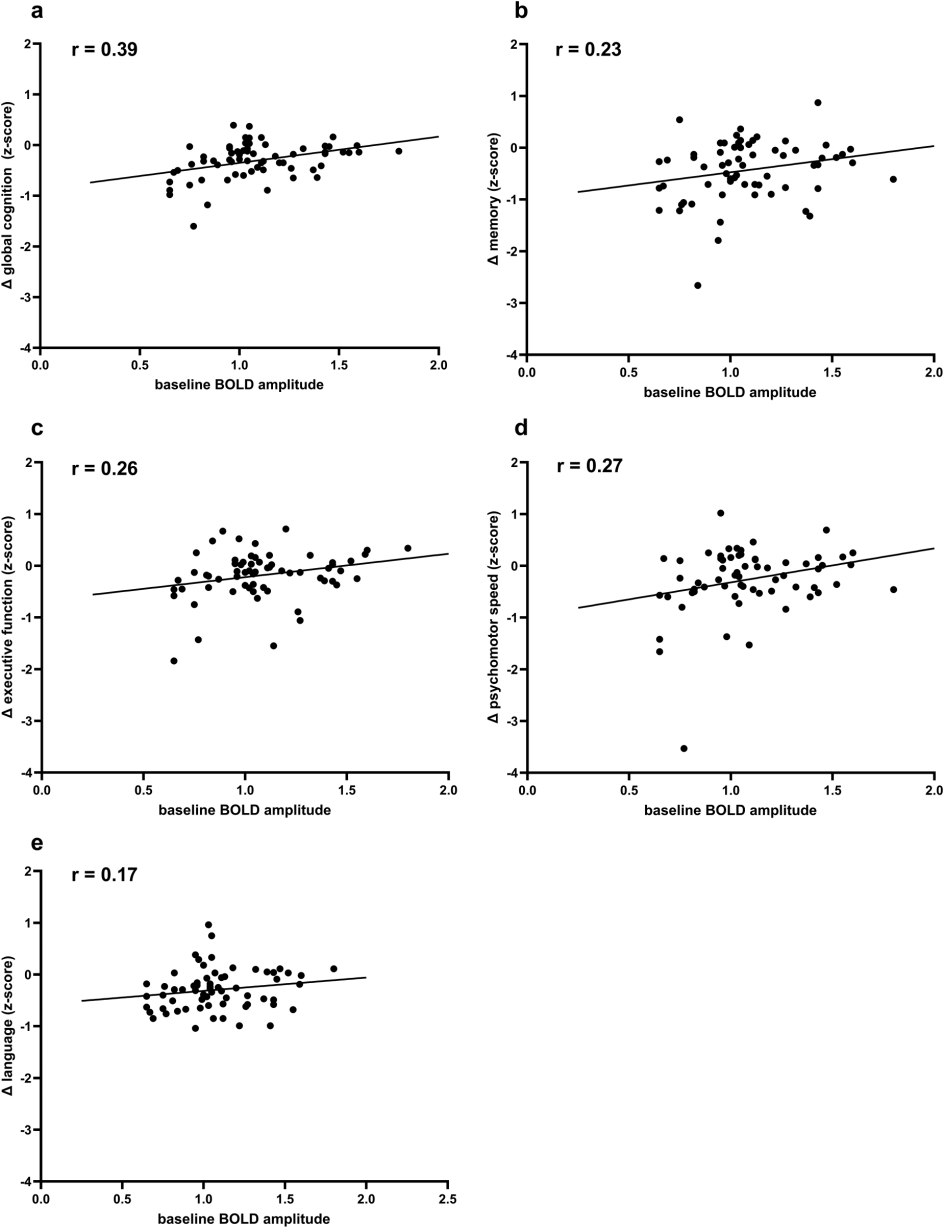
Scatterplots showing the associations between baseline BOLD amplitude and change in cognitive performance over 3.5 years. Pearson's correlation coefficients (r) are displayed in each panel. Lower baseline BOLD amplitude was significantly associated with a greater decline in a) global cognition and c) executive function, but not with b) memory, d) psychomotor speed, or e) language.

Multivariable linear regression analyses assessed the associations between baseline MRI markers and longitudinal changes in cognitive domain z-scores (Table 4), adjusted for age, sex, time interval between assessments, cardiovascular risk factors (hypertension, hyperlipidemia, diabetes, and smoking) and history of stroke. Consistent with the primary analyses, lower baseline BOLD amplitude was associated with greater decline in global cognition (p = 0.012) (Figure 4). Lower baseline BOLD amplitude was also associated with a greater decline in the executive function domain (p = 0.043). No significant associations were observed between baseline BOLD amplitude and domain-specific decline in memory, psychomotor speed, or language (all p > 0.05).

**Table 4.** Associations between baseline MRI markers and delta cognition z-scores.

	Δ global cognition (composite score)			Δ memory			Δ executive function			Δ psychomotor speed			Δ language		
	B (95% CI)	β	p	B (95% CI)	β	p	B (95% CI)	β	p	B (95% CI)	β	p	B (95% CI)	β	p
BOLD amplitude	0.45 (0.11–0.80)	0.34	**0** **.** **012**	0.35 (−0.28–0.97)	0.16	0.271	0.47 (0.02–0.92)	0.27	**0**.**043**	0.49 (−0.21–1.18)	0.20	0.165	0.30 (−0.11–0.71)	0.21	0.146
Lobar MB	0.09 (−0.11–0.28)	0.11	0.370	0.05 (−0.29–0.39)	0.04	0.762	0.23 (−0.01–0.47)	0.23	0.063	0.22 (−0.13–0.57)	0.18	0.205	0.01 (−0.20–0.23)	0.01	0.916
Deep MB	−0.08 (−0.37–0.21)	−0.07	0.582	−0.31 (−0.81–0.19)	−0.16	0.217	0.14 (−0.23–0.51)	0.09	0.458	0.04 (−0.49–0.57)	0.02	0.880	−0.15 (−0.47–0.17)	−0.12	0.345
Lacunar infarct	−0.09 (−0.32–0.14)	−0.10	0.452	−0.05 (−0.44–0.34)	−0.03	0.813	−0.04 (−0.34–0.26)	−0.04	0.791	0.05 (−0.39–0.48)	0.03	0.830	−0.21 (−0.47–0.04)	−0.24	0.098
DPVS-CSO	0.07 (−0.12–0.25)	0.09	0.471	0.14 (−0.16–0.44)	0.12	0.363	0.12 (−0.12–0.35)	0.12	0.328	0.29 (−0.05–0.63)	0.22	0.097	−0.14 (−0.34–0.06)	−0.18	0.164
DPVS-BG	0.10 (−0.10–0.31)	0.13	0.317	0.09 (−0.27–0.45)	0.07	0.616	0.01 (−0.25–0.28)	0.01	0.934	0.30 (−0.07–0.67)	0.23	0.110	0.06 (−0.17–0.29)	0.08	0.585
WMHs	−0.07 (−0.18–0.04)	−0.17	0.203	−0.05 (−0.24–0.14)	−0.07	0.600	−0.06 (−0.20–0.08)	−0.11	0.412	0.02 (−0.18–0.22)	0.03	0.827	−0.11 (−0.23–0.01)	−0.25	0.062

B: unstandardized regression coefficient; β: standardized regression coefficient; CI: confidence interval; BOLD: blood-oxygen-level-dependent; DPVS-BG: dilated perivascular spaces in the basal ganglia; DPVS-CSO: dilated perivascular spaces in the centrum semiovale; WMHs: white matter hyperintensities. Longitudinal associations between baseline MRI markers and change in cognitive domain z-scores were assessed using linear regression analyses. Significant associations are highlighted in bold (p < 0.05).

Among conventional SVD markers, no significant associations were found with domain-specific longitudinal cognitive decline.

## Discussion

This study investigated whether baseline NVC, assessed via BOLD amplitude in response to visual stimulation, is associated with cognitive decline over 3.5 years in individuals ranging from no cognitive complaints to AD-related dementia. We observed that a lower baseline NVC was linked to an increased risk of global cognitive decline, outperforming conventional SVD MRI markers. Our finding supports the view that NVC impairment may serve as an early predictor of future cognitive decline.

Our results build on accumulating evidence that cerebrovascular dysfunction is an important contributor to AD and related cognitive impairment.^41,42^ Prior studies have shown cross-sectional associations between NVC and cognition,^18,20^ but this is, to our knowledge, the first longitudinal study to demonstrate the association with future cognitive decline. Comparable findings have been reported for other measures of microvascular function, such as hypercapnia-induced vasodilation as a proxy for cerebrovascular reactivity, which have been linked to worse cognitive outcomes^43^ and progression to MCI.^44^ Together, these data underscore the role of small-vessel dysfunction in the trajectory of cognitive decline across the disease continuum.

The biological mechanisms underlying this association remain incompletely understood, but several pathways are likely involved. Impaired NVC disrupts dynamic regulation of cerebral blood flow, limiting both the delivery of oxygen and nutrients to neurons^12^ and the clearance of metabolic waste, including Aβ.^45^ These processes may reinforce one another, creating a feedback loop promoting further vascular and parenchymal Aβ accumulation,^46^ neuronal injury, and cognitive decline.

While reduced NVC was associated with cognitive decline in our cohort, most conventional SVD MRI markers, except lacunar infarcts, were not. Previous studies, however, have reported links between SVD markers such as WMHs, DPVS, and lobar microbleeds, and cognitive decline.^8,47–49^ It is conceivable that structural markers primarily detect late-stage, cumulative injury, and individuals with extensive SVD may already have poor cognitive performance, which limits the predictive range of these markers. In contrast, NVC, as a functional measure, may capture more subtle and earlier microvascular deficits across a broader range of cognitive performance.

We did not observe a significant association between baseline NVC and domain-specific cognitive decline, except for executive function, which may reflect limited statistical power, attrition bias due to drop-out of participants with more severe decline, or the occipital focus of our stimulation paradigm. Although occipital NVC is thought to reflect global vascular responsiveness, it is important to note that certain brain regions, especially the hippocampus, may be more susceptible to NVC impairment.^50^

An important limitation to our study is the modest sample size, which may limit generalizability of our findings. In addition, participants with cognitive decline were older, and although age was included as a covariate, residual age-related effects cannot be entirely excluded given the limited sample size. Moreover, intracranial arterial disease could not be evaluated because vascular imaging data required for this assessment were unavailable. As such pathology may contribute to cerebral hypoperfusion and cognitive decline, future studies incorporating these measures are warranted.

Despite these limitations, our findings suggest that BOLD amplitude reflects early brain alterations associated with cognitive decline and may provide complementary information beyond conventional structural SVD MRI markers. At present, its most plausible application lies in research and trials settings, where visually stimulated BOLD measures are already used as outcome marker in clinal CAA trials.^51^ In this context, BOLD amplitude could further contribute to risk stratification or serve as a covariate to account for expected cognitive decline. While BOLD amplitude may offer additional insight in clinical settings where MRI is already routinely acquired, further validation in larger, longitudinal, and multicenter cohorts are required before any clinical application can be considered.

Future studies should therefore include larger, multicenter cohorts, particularly enriched for early disease stages, to validate NVC as a prognostic marker and potential therapeutic target in dementia. Expanding NVC assessment to additional brain regions, such as the hippocampus and prefrontal cortex, could clarify region-specific vulnerability. Moreover, multimodal studies combining NVC with Aβ and tau imaging may further disentangle the interplay between vascular dysfunction and neurodegenerative pathology.

In conclusion, our findings suggest that baseline NVC is associated with future global cognitive decline and appears to outperform most conventional structural SVD MRI markers. These results support the potential utility of NVC as a prognostic marker of cognitive decline in preclinical and early-stage AD. By capturing functional vascular deficits that precede structural damage, NVC may offer a valuable window for early intervention and monitoring of therapeutic strategies aimed at preserving cognitive function. Overall, these results underscore the critical role of cerebrovascular health in cognitive trajectories and support further investigation of NVC in larger, longitudinal studies as a prognostic tool and potential target for intervention.

## Supplemental Material

sj-docx-1-alz-10.1177_13872877261427735 - Supplemental material for Neurovascular decoupling: An early indicator of cognitive decline Supplemental material, sj-docx-1-alz-10.1177_13872877261427735 for Neurovascular decoupling: An early indicator of cognitive decline by Suzanne E. van Dijk, Nadieh Drenth, Anne Hafkemeijer, Isa Draaijer, Gerda Labadie, Marie-Noëlle W. Witjes-Ané, Gerard J. Blauw, Serge A. R. B. Rombouts, Jeroen van der Grond and Sanneke van Rooden in Journal of Alzheimer's Disease
