# Supplementary material for: Neurovascular decoupling: An early indicator of cognitive decline
Source: J Alzheimers Dis. 2026 Mar 13;110(4):1899–912. doi: 10.1177/13872877261427735 (PMC13058159; doi:10.1177/13872877261427735)
Supplement: sj-docx-1-alz-10.1177_13872877261427735 - Supplemental material for Neurovascular decoupling: An early indicator of cognitive decline [file sj-docx-1-alz-10.1177_13872877261427735.docx]

**Supplemental Material**

**Neurovascular decoupling: An early indicator of cognitive decline**

**N=47**

**No cognitive complaints** participated in baseline study

**N=33**

**MCI** patients participated in baseline study

**Reasons non participation follow-up:**

2 deceased

4 with severe cognitive disorders / residing in nursing homes

2 with (severe) somatic conditions

1 did not have MRI at baseline

5 expressed no interest

1 unknown/other reasons

1 did not respond

**Reasons non participation follow-up:**

1 deceased

2 with severe cognitive disorders / residing in nursing homes

3 with (severe) somatic conditions

2 expressed no interest

2 unknown/other reasons

1 did not respond

**N=17**

**MCI diagnosis at baseline p**articipated in follow-up study

**N=17**

**SCI** **diagnosis at baseline** participated in follow-up study

**N=36**

**No cognitive complaints at baseline** participated in follow-up study

**Reasons non participation follow-up:**

2 with (severe) somatic conditions

1 did not have MRI at baseline

2 expressed no interest

3 unknown/other reasons

3 did not respond

**N=28**

**SCI** patients participated in baseline study
